# Building bridges between a community and an academic medical center via community tours

**DOI:** 10.1017/cts.2020.7

**Published:** 2020-01-20

**Authors:** Megan B. Irby, Keena R. Moore, DeWanna Hamlin, Olivia Brown, Grisel Trejo, Phillip Summers, Stephanie Daniel, Joseph A. Skelton, Michael Lischke, Scott D. Rhodes

**Affiliations:** 1 Maya Angelou Center for Health Equity, Wake Forest School of Medicine, Winston-Salem, NC, USA; 2 Program in Community Engagement, Wake Forest School of Medicine, Winston-Salem, NC, USA; 3 Department of Family and Community Medicine, Wake Forest School of Medicine, Winston-Salem, NC, USA; 4 Department of Family and Community Medicine, and an associate director of the Program in Community Engagement, Wake Forest School of Medicine, Winston-Salem, NC, USA; 5 Department of Pediatrics, and an associate director of the Program in Community Engagement, Wake Forest School of Medicine, Winston-Salem, NC, USA; 6 Department of Family and Community Medicine, Wake Forest School of Medicine, and the Richard Janeway Distinguished Director of the Northwest Area Health Education Center, Winston-Salem, NC, USA; 7 Department of Social Sciences and Health Policy, and the director of the Program in Community Engagement, Wake Forest School of Medicine, Winston-Salem, NC, USA

**Keywords:** Community tour, community engagement, social determinants of health, trust, community–academic partnership, translational research

## Abstract

Academic medical centers (AMCs) face challenges in conducting research among traditionally marginalized communities due to long-standing community mistrust. Evidence suggests that some AMC faculty and staff lack an understanding of the history of distrust and social determinants of health (SDH) affecting their communities. Wake Forest Clinical and Translational Science Institute Program in Community Engagement (PCE) aims to build bridges between communities and Wake Forest Baptist Health by equipping faculty, clinicians, administrators, and staff (FCAS) with a better understanding of SDH. The PCE collaborated with community partners to develop and implement community tours to improve cross-community AMC understanding and communication, enhance knowledge of SDH, and build awareness of community needs, priorities, and assets. Nine day-long tours have been conducted with 92 FCAS. Tours included routes through under-resourced neighborhoods and visits to community assets. Participant evaluations assessed program quality; 89% reported enhanced understanding of access-to-care barriers and how SDH affect health; 86% acknowledged the experience would improve future interactions with participants and patients; and 96% agreed they would recommend the tour to colleagues. This work supports the use of community tours as a strategy to improve cross-community AMC communication, build trust, and raise awareness of community needs, priorities, and assets.

## Introduction

The presence of health disparities is well established and deeply rooted in the social, economic, and environmental contexts in which people live [1,2], and the impact of these factors on health is most pronounced among marginalized groups, including racial/ethnic, sexual, and gender minorities, and among economically disadvantaged communities [3]. Many disparities are driven by social determinants of health (SDH), or “the structural determinants and conditions in which people are born, grow, live, work and age… shaped by the distribution of money, power and resources at global, national and local levels”[4,5], including socioeconomic status, education, the physical environment, employment, social support networks, and access to health care. SDH may also include historical marginalization that has led to broad mistrust within communities in regard to medical and health care institutions. However, health disparities and SDH often are under-explored in the medical curricula [6-8], which likely is a barrier hindering how well health care institutions can understand and care for the communities they seek to serve.

Examination of SDH is a first step to equipping medical faculty and staff with fundamental knowledge to better meet the needs of their research participants and patients [8,9]. Understanding SDH may help to develop and strengthen relationships between investigators, care providers, and the communities they serve. As a result, by raising the level of consciousness about SDH, it may also be possible to enhance understanding of and appreciation for the historical, social, and economic factors that influence research participants and patients’ lives, their participation in research and utilization of the health care system, and their ability and comfort levels with partnering with care providers to make decisions about their own health. Oppositely, neglecting to consider SDH may worsen systemic inefficiencies and ineffectiveness in the promotion of health, prevention of disease, and the provision of health care.

Like many academic medical centers (AMCs), Wake Forest Baptist Health (WFBH) historically has faced challenges in engaging with the community and meeting the needs of marginalized populations due to a legacy of historical and institutionalized racism and ongoing community mistrust of the health care system and medical research [10-12]. The African American community has been a notable victim of injustices from the institution throughout the years, as the history of health care for the African American community in Winston-Salem is one characterized by marginalization [13-16]. Like many medical institutions and governments, WFBH and Winston-Salem played a large role in supporting and implementing discriminatory practices during the Jim Crow era.

From 1933 to 1974, WFBH, like many institutions, participated in the North Carolina state eugenics program [16,17]. Of the more than 7600 forced sterilizations sanctioned by the North Carolina Eugenics Board, the vast majority were imposed on African American and/or mentally ill patients [14,16]. WFBH has attempted to learn from and repair the damage of its history, but the wounds of the past still exist within the communities that surround it. The events of the past have transcended into and continue to hinder present relationships between the community and the medical center, and are reflective of the same strained community–academic relationships in communities across the USA [18].

In addition to WFBH’s involvement in the state eugenics program, tensions between the African American community and the city of Winston-Salem were further exacerbated by the construction of Highway 52 in the 1950s, an urban renewal or slum clearance project that bisected the city and largely segregated its populations [19]. Known informally among residents as the most racist physical structure in Winston-Salem [19], Highway 52 cuts through Winston-Salem’s “black and brown neighborhoods” isolating their vital resources, including access to quality medical care. The Kate Bitting Reynolds Hospital for African Americans was the only source of health care available to Winston-Salem’s African American community for most of the 20th century, but the closure of the hospital in 1970 left many African Americans with even fewer opportunities for health care as the larger and primarily white hospital, WFBH, was unprepared, and in some cases unwilling, to serve African American patients [19]. These atrocities and injustices continue to survive in the living memories of Winston-Salem’s African American communities and decry the need for improved community–AMC relationships.

Here, we detail an innovative approach to enhance medical professionals’ understanding of SDH and their local communities through our development and implementation of a community tour. Through the community tour, WFBH’s Program in Community Engagement (PCE) provides education for providers, administrators, and researchers to better understand how traditionally marginalized communities surrounding WFBH have been isolated, discriminated against, and withheld from accessing health resources over time. Through increased understanding of the community and its historical context, we aim to enhance and nurture engagement among medical professionals and the community surrounding the AMC, as WFBH seeks to provide and deliver equal access to care for all persons. The community tour is intended to be a bold source of cultural change within the institution, as it encourages intentional inclusion of and meaningful engagement with the community going forward.

## Materials and Methods

Community tours are a strategy to address inequality and social factors that affect community health by opening up doors between AMCs and the community, and creating opportunities for faculty and staff to learn about and engage in dialog with community organizations around SDH, foster relationships with the community, and understand community needs, priorities, and assets. Our community tour aims to help medical faculty and staff better understand the SDH directly affecting their community, foster trust and connections between the institution and these organizations, and encourages them to reflect on the roles they play as investigators and care providers who shape the health of communities.

Numerous studies detail the use of training programs to enhance understanding of SDH among medical professionals, and to this end, it is not uncommon for AMCs to offer “windshield tours” or “community plunges” [20]. To our knowledge, however, there are no guidelines for implementing SDH education for medical faculty and staff, and no programs have offered similar community tour opportunities beyond a medical student education setting.

## Community Tour Development and Implementation

**Fig. 1.**
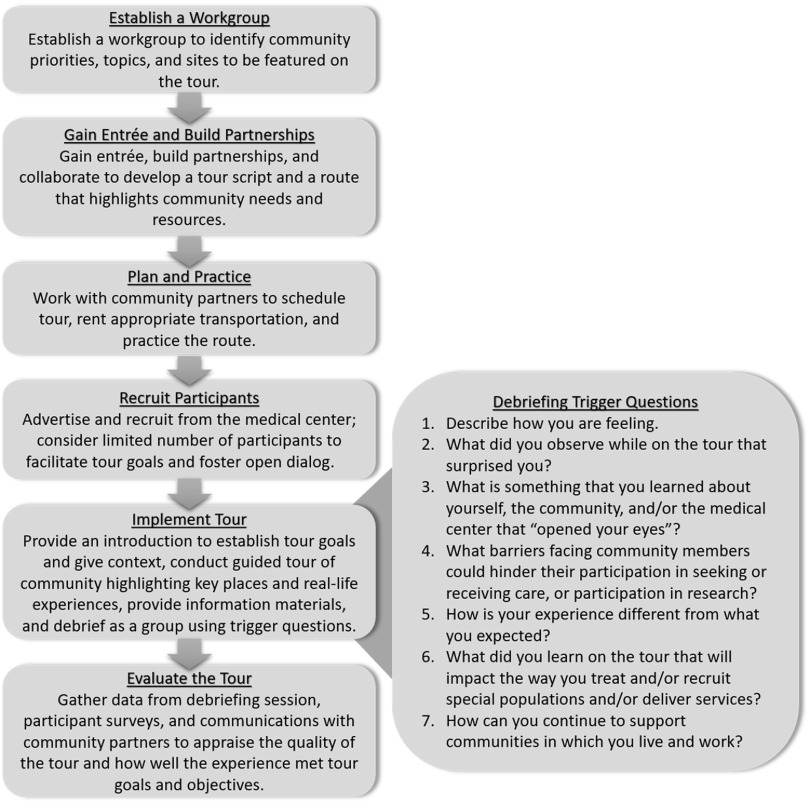
Community tour process.

A six-step process was used to plan and implement the tour with involvement from community partners during each of the six steps (Fig. 1):

1. **Establish a Workgroup:** Following a community tour program developed for medical residents by WFBH’s Northwest Area Health Education Center[21], workgroup was established including community members, medical faculty and staff, and members from the PCE’s stakeholder advisory committee (representatives from more than 40 community organizations that provide ongoing guidance and feedback to the Wake Forest Clinical and Translational Science Institute). Based on recent community health needs assessments such as the State of the County Health Report [22] and existing community data for the African American community, the workgroup collectively prioritized SDH with the most salience to the local community, and determined topics and sites to be featured on the tour (Table 1).
2. **Gain Community Entrée and Build Partnerships**: The workgroup identified potential community partners and established relationships. Each tour was designed to take place within the city of Winston-Salem with the intention of raising consciousness of SDH and health disparities among WFBH faculty and staff, while also featuring key community resources and partners. Workgroup members worked with community partners to create tour routes that intentionally highlighted areas of interest pertinent to the community priorities and assets determined by the workgroup, and to encourage reflection on health needs and the historical context of the community. The workgroup also developed and refined a semi-structured tour script and identified potential locations where participants could stop and exit along the tour route, meet community partners, and learn about specific community assets and resources. For each tour, four to five partnering community organizations agreed to participate to host brief presentations highlighting their history and the services they provide, answer questions, and provide a tour of their facilities.
3. **Plan and Practice**: Over the course of a 4-month planning period with weekly workgroup meetings, full-day (8 hour) community tours were fully scheduled and scripted. PCE staff procured a 12-person bus, and pre-drove the tour route to determine route timing of all stops and exits. PCE staff met with community partners to nurture relationships, plan messaging and structure, and prepare for a thoughtfully planned and well-timed tour.
4. **Recruit**: Participants were recruited from the medical center via various electronic communications and word-of-mouth. The target population included investigators, care providers, and staff with an interest in learning more about the communities they serve, increase understanding of how SDH affect study participants and patients, and increase inclination to craft research to better integrate special populations into their research. Recruitment for each tour was intentionally limited to 10–12 participants to ensure rich discussion and to limit the need for a larger tour bus, which likely would be viewed as intrusive or voyeuristic within the neighborhoods featured on the tour.
5. **Implement the Tour**: Each tour featured an introduction session, a guided tour of the community including “stops and exits” at various locations of interest, lunch at a community organization, and a post-tour debriefing session.
  *Introduction (1 hr.)* – Prior to embarking on the tour, breakfast was provided for tour participants at the PCE office. PCE staff met with participants to provide background information about the tour, present information about the history of the community and past and current relationships between the community and the medical center, and to provide an overview of local SDH and health disparities. To provide context for and help define the purpose of the community tour, participants viewed “Roadways and Foodways: Finding Our Way Home,” a 20-minute documentary created by local documentarians (https://youtu.be/qHCvXhn8oLY) [23]. Through oral histories from local residents, the film describes community change over time, the consequences of the 1960s urban renewal of Winston-Salem, and how construction of the interstate system disrupted thriving African American neighborhoods and damaged the local food environment along with existing businesses and social networks.
  *Tour with Lunch (6 hr.)* – Two PCE staff served as tour guides highlighting community resources, assets, and areas of historical significance. Tour guides not only held a strong knowledge of SDH, but also of the community, as both are long-time residents of the Winston-Salem community that are members of the marginalized group with a robust personal understanding of the community’s history, health needs, social issues, and assets. During the tour, the guides shared stories with participants to illustrate the true-to-life experiences of community members (e.g., the barriers that a mother of two faces when attempting to go to the medical center, health department, or pharmacy; and the challenges families experience when attempting to access nutritious foods when the closest grocery store is 7 miles away and they have no reliable transportation). Through these stories, the guides encouraged participants to personally reflect on and engage in dialog with one another about what they see, hear, and experience throughout the tour.
  Each participant received a binder with additional information about community demographics, SDH, health disparity and asset mapping, and community highlights. Each tour featured approximately five stops and exits where participants engaged with community partners at each location. Participants were provided lunch at a community location (e.g., a restaurant run by a local food bank that offers food service job training). During lunch, community leaders and representatives from community organizations and those affected by the history of the institution, including a former and current administrator associated with WFBH, give brief presentations describing their roles in the community, their interactions and work with the institution as those in the marginalized populations, provided historical context for the tour, such as exploring the history of eugenics and the effects of Highway 52 on the community, and highlighted community resources.
  *Debriefing (1 hr.)* – Following the tour, participants returned to the PCE office and PCE staff engaged in empowerment-based facilitation to lead a discussion to guide participants in systematically processing their experiences in safe space through *reflection-in-action*, a process tool that encourages reflection on a new experience while that experience is still ongoing [24,25]. To do so, trigger questions were used to move from concrete to more abstract thinking, raise consciousness about SDH, and foster critical dialog (Fig. 1). The goal of debriefing sessions was to create an opportunity of introspection about SDH, participants’ own stories and cultures, and to address seemingly uncomfortable topics (e.g., race, power, and privilege).
6. **Evaluate the Tour**: Qualitative data were collected from participants during the debriefing session. Additionally, all participants were invited via email to complete an electronic survey describing their experiences with the tour, their perceptions of the information presented, and the value of the overall tour for their professional work. This provided an opportunity for reflection-*on*-action [24,25], a process which encourages participants to reflect on how their own behaviors and practices can be developed after gaining new knowledge, and how they might incorporate what was learned from the tour into their practice as investigators and care providers. PCE staff also visited community partners to thank them for participation and to elicit their feedback. To monitor the extent to which tour experiences influence participant attitudes and practice, evaluation surveys are distributed 6-month post-tour. All information collected from participants informed planning for future tour iterations.

**Table 1.** Community tour highlights, stops, and exits

SDH	Community tour highlights, stops, and exits
Historical context	Describe the history of the community and identify areas of historical significance (e.g., landmarks, business, cemeteries, and neighborhoods) to help participants gain an understanding of the context in which SDH affect local community populations.
Income	Describe disparities in income, and availability and access to organizations that seek to address income disparities (e.g., community action agencies, social services, food/clothing assistance programs, food pantries, and job training programs). Discuss the roles and impact of these organizations, and challenges they face in supporting their communities and patrons.
Food and nutrition	Identify local food deserts and the availability and locations of local markets and grocery stores. Discuss the politics of grocery store placement and differences in food options/pricing between communities and across types of stores (i.e., super markets versus corner stores and gas stations).
Education	Describe the history of education-related political issues and differences in resource allocation to local schools. Identify and describe the history, location, and demographics of schools (e.g., primary, trade, and institutions of higher learning).
Health care	Identify and describe the availability and accessibility of primary care offices, community clinics, dental offices, community pharmacies, mental health services, school-based health programs, and emergency medical assistance services. Discuss differences in health care access and utilization within the context of history and social determinants of health.
Neighborhoods and built environments	Describe the demographics of communities and history of segregation; identify and describe income-based housing and neighborhoods, the impact of roadways and road conditions on communities, and the walkability of neighborhoods (i.e., presence of sidewalks, bike lanes, and bus stops) and proximity to health care resources and nutritious foods. Identify and describe the significance of community centers, greenways, parks, local attractions, and sports complexes.
Transportation	Describe transportation policies, the availability and reliability of public transportation, and accessibility to bus stops and routes. Discuss the challenges of accessing and utilizing public transportation, and the role of transportation in promoting health.
Social cohesion	Identify and describe organizations and businesses that provide opportunities for fellowship and foster an appreciation for local culture, history, arts, and education (e.g., cultural arts centers, religious institutions, and faith-based groups). Discuss the role of these organizations in promoting resilience and social cohesion within and across communities.

SDH, social determinants of health

## Results

To date, 10 tours have been completed with a total of 104 participants, including a variety of health professionals from diverse departments (Table 2), and 73 have completed follow-up surveys. Tours are held twice each year; once in the Spring and once in the Fall.

**Table 2.** Participants and departmental affiliations

Participants	N = 92	Examples of departments/affiliations
Academic faculty	23	Social Sciences and Health Policy, Epidemiology & Prevention, Clinical Informatics, Anesthesiology
Physician	20	Family & Community Medicine, Gerontology, Internal Med, Orthopedics, Obstetrics and Gynecology, Pediatrics, Cancer/Oncology, Emergency Medicine
Admin	8	Hospital Administration, Development & Alumni Affairs, Medical Education, Ambulatory Care, FaithHealth, HR Adminstration
Staff*	41	Various depts. (*Clinic coordinators, nurses, patient navigators, program managers, research staff)

During the debriefing sessions (Table 3), participants overwhelmingly reported that they had gained a deeper understanding of the challenges participants and patients face in leading healthy lives and the difficulties they faced navigating the health care system (e.g., access to resources and access-to-care and transportation issues). Many participants reported that this new knowledge of the local community and SDH would influence their own professional practice (e.g., greater flexibility for participants and patients who arrive late for appointments due to relying on public transit).

**Table 3.** Key findings from debriefing sessions

Debriefing question	Findings and key quotes
What did you learn on the tour that will impact the way you treat/recruit special populations and/or deliver services?	It is important to instill knowledge of health equity and social determinants of health in the medical student curriculum “Medical providers should develop a deeper appreciation of other peoples’ experiences.” It is important to expand our worldviews and have deeper connection with others It is imperative that medical providers nurture trust Medical providers must take time to learn community needs
What is something you learned about yourself, community, and/or organizations that opened your eyes?	Formerly thriving businesses and business owners were left isolated and discouraged; their livelihoods were taken away, repeatedly over time, due to racism, gentrification, and big business. Communities were destroyed and resources continue to dwindle to this day. “There is so much I didn’t know…how do I learn more?” “There is potential for additional collaboration in a sustainable way.”
What did you observe that surprised you?	Many participants did not fully understand the reputation of WFBH in relation to sterilization and other historical abuses committed by the medical system. The video portrays the history of division in the local community that continues to persist decades later.
Describe how you are feeling?	“Tired but motivated to share with learners: med students, residents, and emergency department folks.” “Motivated to get involved in the local community, my own community.” “Enlightened, embarrassed, and saddened by the level of disenfranchisement.” Uplifted, positive, inspired to move forward Frustrated and conflicted by the history and how the community is still being impacted Humility

Post-tour surveys were administered via email and REDCap (Research Electronic Data Capture, a secure web application for building and managing online surveys and databases) to assess program quality. Survey results indicated that 89% of participants reported enhanced understanding of access-to-care barriers and how SDH impact health; 86% reported to have gained a better understanding of the environments in which their study participants and patients live and how those environments impact health; 86% acknowledged that the tour experience would impact interactions with participants, patients, and future research study designs; and 96% reported that they would recommend the tour to colleagues. Feedback from community partners and participants guided quality improvements with each tour iteration.

As a result of this work, other medical center departments have adopted this community tour model to enhance medical education among medical students, residents, and research staff (e.g., research program managers, study coordinators, and research assistants).

## Discussion

With each tour iteration, the PCE staff and tour guides continue to identify opportunities to enhance the tour. The lessons learned described below will continue to shape the evolution of future community tours.

It is important to recognize that though each tour is built on the same learning objectives, each tour is unique as participants bring with them their own lived experiences and the ever-changing sociopolitical landscape affords nuanced discussion. Thus, the tour guides and planning team are flexible and understand that each new tour will not be an exact replica of those that came before. Furthermore, community partners including stops and speakers may differ across tours.

Although there has been a focus on researchers’ and clinicians’ potential change in practice, there has also been the inclusion of participants representing other roles within the institution, such as marketing and communications. Our aim is that other institutional departments will consider their tour experience when crafting content, both internal and external, that represents the institution, and communicates messaging to communities in ways that are respectful, culturally sensitive and appropriate, and are inclusive of all.

Each tour is intentionally designed to raise consciousness about unique local SDH and health disparities while also highlighting community assets. This balance is useful for resolving preconceived notions and stereotypes about the local community and its residents, and also educates participants about the resources and partners in the community that are well poised to address patient health issues outside of the walls of the medical center. Paired with “real-life” examples and critical discussions, the tour experience encourages participants to reflect on their roles as investigators and care providers and how they might transform their professional practices to better address research study participation and patient health. Tours for different communities will address different communities and different needs. There must be consideration and discussion with community to understand the needs that are most important to demonstrate.

Limiting tour participation to no more than 12 participants allows tour guides to not only cultivate an atmosphere of trust and foster open dialog, but also is done out of respect for community members, as taking large tour groups through the community neighborhoods could be perceived as voyeuristic. The PCE also offers additional opportunities for participants to take a deeper dive into community-engaged research approaches (e.g., consultations, continuing education programs, and pilot funding), as a true understanding of SDH and health equity cannot be achieved through a one-time tour experience [26].

Despite high demand for tours, the PCE has capacity to offer tours twice each year. In our experience, we have learned that the frequency of tours should be based on a number of factors, including: ability to recruit an adequate number of participants, capacity of program staff and tour facilitators, the capacity of participating community partners, changes in community schedules by season, transportation logistics and roadwork schedules, venue availability, availability of key presenters, timing of other community events and competing institutional commitments, and financial resources.

Having a well-timed tour script, pre-driving the route, meeting with community hosts, and anticipating potential delays are essential prior to the tour date. Timekeeping on the day of the tour also is crucial, as encountering delays has potential to impact participants’ experiences, the amount of time allotted for stops and exits, and whether community partners continue to participate. Throughout the tour, there is a conscious effort to allow flexible opportunities for paired or small group discussion during travel points, at lunch, and during stops at community sites. Unplanned, or spontaneous discussion, rapport building and personal connection with participants and community partners are encouraged.

It is important to nurture relationships with community partners and to regularly identify and interact with new and existing community resources that potentially could be featured on the tour. A comprehensive and up-to-date awareness of community assets may be useful for participants who wish to share such information with their patients and research participants. Future directions for this work include disseminating findings to, and eliciting feedback from, patients, the lay community, and among care providers and administrators. We also aim to capture long-term outcomes among patients, community partners, and past tour participants to better understand the broader impact of community tours on relationships between the community and the AMC.
